# Lichen scléro-atrophique extra-génital: à propos d’un cas

**DOI:** 10.11604/pamj.2016.25.190.10509

**Published:** 2016-11-24

**Authors:** Youssef Zemmez, Mohammed El Amraoui, Ahmed Bouhamidi, Jaouad El Azhari, Nadia Ismaili, Laila Benzekri, Mariame Meziane, Badreddine Hassam, Karima Senouci

**Affiliations:** 1 Service de Dermatologie, CHU Ibn Sina Rabat, Maroc

**Keywords:** Lichen scléro-atrophique, atteinte cutanée, histologie, Lichen sclerosus et atrophicus, cutaneous involvement, histology

## Abstract

Le lichen scléro-atrophique est une dermatose inflammatoire d'évolution chronique, avec un tropisme particulier pour les muqueuses génitales externes, l'atteinte cutanée isolée reste rare. Nous rapportons un cas de lichen scléro-atrophique chez une femme avec atteinte purement cutanée soulignant ainsi l'intérêt d'évoquer ce diagnostic en absence d'une atteinte génitale évidente.

## Abstract (en)

Lichen sclerosus et atrophicus is a chronic inflammatory dermatosis, characterized by a specific external genital mucosal tissue tropism. Isolated cutaneous manifestation is rare. We report a case of lichen sclerosus et atrophicus in a woman with purely cutaneous involvement, in order to highlight the importance of evoking this diagnosis in the absence of obvious genital involvement.

## Introduction

Décrit en 1887 par Hallopeau, le lichen scléreux ou scléro-atrophique (LSA) est une dermatose inflammatoire fibrosante d´évolution chronique et de prédominance féminine, touchant surtout la région ano-génitale (80%). La localisation purement extra-génitale ne se voit que dans 2,5% des cas.

## Patient et observation

**Figure 1:**
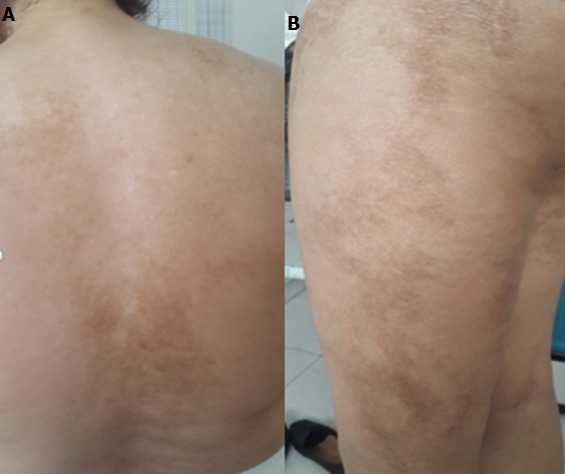
A) plaques blanchâtres atrophiques au niveau du dos; B) au niveau des cuisses

Mme A.N âgée de 45 ans, mariée et mère de 3 enfants, ayant comme antécédents: une tuberculose ganglionnaire en 1997, traitée par anti-bacillaires pendant 09 mois, consultait pour des lésions blanchâtres diffuses, modérément prurigineuses évoluant depuis 07 mois, intéressant au début le dos, le tronc puis extension aux membres supérieurs et membres inférieurs, avec installation plus tard d'une sclérose cutanée diffuse. L´examen clinique montrait des plaques de couleur blanc -nacré et atrophiques, confluentes en larges placards par endroits au niveau du dos, du tronc, des membres supérieurs ainsi que les membres inferieurs (Figure 1). Le reste de l´examen clinique, notamment cutanéo-muqueux et général, était normal.

**Figure 2:**
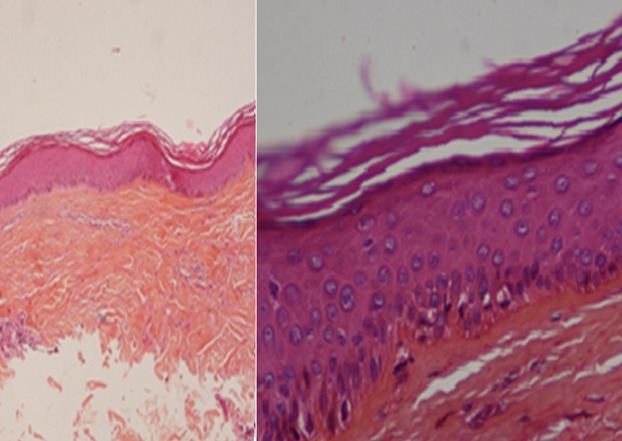
Coloration HES: atrophie épidermique avec un infiltrat inflammatoire interstitiel du derme moyen

**Figure 3:**
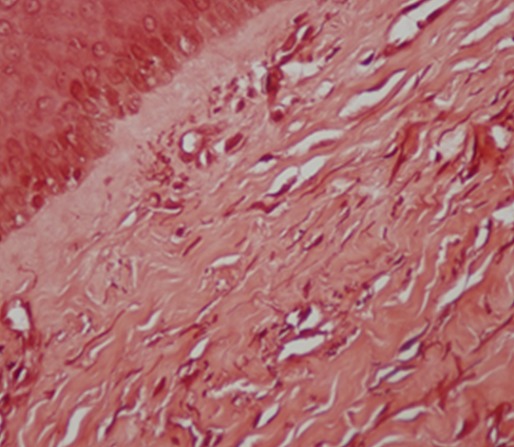
Coloration à l’orceine: derme papillaire déshabité des fibres élastiques, épaissies au niveau du derme moyen

**Figure 4:**
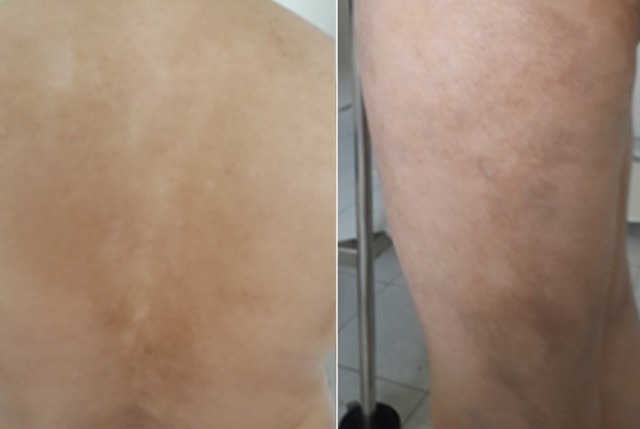
Régression partielle des plaques atrophiques

L´étude histologique montrait un épiderme atrophique, siège d´une hyperkératose orthokératosique avec par endroit une hyperkératose ostio-folliculaire ainsi qu´un derme papillaire œdémateux et un derme profond fibreux comportant un infiltrat inflammatoire lympho-histiocytaire (Figure 2). La coloration spéciale par l´orcéïne objectivait une déshabitation du derme papillaire des fibres élastiques qui sont rejetées en Profondeur (Figure 3). Le diagnostic de lichen scléro-atrophique extra-génital était retenu. Le bilan biologique comprenant les sérologies de l´hépatite B et C était normal. Un traitement à base d´émollients et d´UVB-thérapie a été préconisé. L´évolution était marquée par l´amendement du prurit et l´amélioration partielle des lésions après 8 séances d'UVB-thérapie (Figure 4).

## Discussion

Le lichen scléreux ou scléro-atrophique (LSA) est une dermatose inflammatoire fibrosante d´évolution chronique et de prédominance féminine, touchant surtout la région ano-génitale (80%) [1]. La localisation purement extra-génitale ne se voit que dans 2,5% des cas [2]. La physiopathologie ferait intervenir plusieurs facteurs: hormonaux, génétiques (HLA II DQ7), infectieux (Borrelia burgdorferi) ou traumatiques par phénomène de Köebner [2].

Cliniquement, les lésions se présentent sous forme de plaques blanchâtres ou de couleur blanc nacré, « porcelainées», atrophiques, intéressant surtout le tronc, la racine des membres et les plis. Le prurit est inconstant. Des formes cliniques blaschkolinéaire et bulleuse ont été décrites [3, 4]. Le diagnostic repose sur l´histologie cutanée qui révèle une atrophie de l´épithélium malpighien avec horizontalisation de la basale, une hyperkératose folliculaire, et surtout la présence d´une bande sous épithéliale faite de collagène fibreux ou œdémateux au niveau du derme superficiel, dépourvue de fibres élastiques à la coloration à l´orcéïne [5].

Le traitement du LSA extra-génital n´est pas codifié et fait recours à plusieurs thérapeutiques: dermocorticoïdes de classe très forte, antipaludéens de synthèse, tacrolimus, pimécrolimus, rétinoïdes, avec des résultats satisfaisants sous calcipotriol et photothérapie UVB [6, 7]. Sur le plan pronostique, et pour les formes de l'adulte, les atteintes cutanées posent essentiellement un problème esthétique par leur évolution chronique, leuco-dermique et atrophique. Certaines lésions peuvent devenir bulleuses et s'ulcérer. Des proliférations mélanocytaires, nævus et mélanomes peuvent se développer sur les lésions de LSA [8]. Contrairement au lichen scléreux génital, le LSA extra-génital se complique rarement de transformation maligne. Ceci pourrait s´expliquer par la diminution de l´expression du marqueur Ki67 et du p53 au cours du LSA [9].

## Conclusion

L'atteinte cutanée du LSA pose essentiellement un problème esthétique par son évolution chronique, leuco-dermique et atrophique. Contrairement au lichen scléreux génital, le LSA extra-génital se complique rarement de transformation maligne.

## References

[1] Tasker GL, Wojnarowska F (2003). Lichen sclerosus. Clin Exp Dermatol..

[2] Ballester I, Bañuls J, Pérez-Crespo M, Lucas A (2009). Extragenital bullous lichen sclerosus atrophicus. Dermatol Online J..

[3] Choi SW, Yang JE, Park HJ, Kim CW (2000). A case of extragenital lichen sclerosus following Blaschko's lines. J Am Acad Dermatol..

[4] Khatu S, Vasani R (2013). Isolated localised extra-genital bullous lichen sclerosus et atrophicus: a rare entity. Indian J Dermatol..

[5] Cavelier-Balloy B (2012). Lichen scléreux. Ann Dermatol Venereol..

[6] Colbert RL (2007). Progresive extragenital lichen sclerosus successfully treated with narrowband UVBphototherapy. Arch Dermatol..

[7] Kreuter A (2002). Extragenital lichen sclerosus successfully treated with topical calcipotriol: evaluation by in vivi confocal laser scanning microscopy. Br J Dermatol..

[8] Carlson JA (2002). Arch. Dermatol..

[9] Scurry J, Whitehead J, Healey M (2001). Histology of lichen sclerosus varies according to site and proximity to carcinoma. Am J Dermatopathol..

